# Different hydrogen-bonded chains in the crystal structures of three alkyl *N*-[(*E*)-1-(2-benzyl­idene-1-methyl­hydrazin­yl)-3-hy­droxy-1-oxopropan-2-yl]carbamates

**DOI:** 10.1107/S2056989015010440

**Published:** 2015-06-06

**Authors:** Thais C. M. Noguiera, Alessandra C. Pinheiro, James L. Wardell, Marcus V. N. de Souza, Jordan P. Abberley, William T. A. Harrison

**Affiliations:** a Fundação Oswaldo Cruz, Instituto de Tecnologia em Fármacos–FarManguinhos, Rua Sizenando Nabuco, 100, Manguinhos, 21041-250 Rio de Janeiro, RJ, Brazil; b Department of Chemistry, University of Aberdeen, Meston Walk, Aberdeen AB24 3UE, Scotland

**Keywords:** Carbohydrazide, methyl­ation, hydrogen bonds, chain, crystal structure

## Abstract

The crystal structures of three methyl­ated hydrazine carbamate derivatives prepared by multi-step syntheses from l-serine are presented, namely benzyl *N*-{(*E*)-1-[2-(4-cyanobenzylidene)-1-methylhydrazinyl]-3-hydroxy-1-oxopro­pan-2-yl}carbamate, C_20_H_20_N_4_O_4_, *tert*-butyl *N*-{(*E*)-1-[2-(4-cyanobenzylidene)-1-methylhydrazinyl]-3-hydroxy-1-oxopropan-2-yl}carbamate, C_17_H_22_N_4_O_4_, and *tert*-butyl *N*-[(*E*)-1-(2-benzylidene-1-methylhydrazinyl)-3-hydroxy-1-oxopro­pan-2-yl]carbamate, C_16_H_23_N_3_O_4_. One of them shows that an unexpected racemization has occurred during the mild-condition methyl­ation reaction. In each crystal structure, the mol­ecules are linked into chains by O—H⋯O hydrogen bonds, but with significant differences between them.

## Abstract 2

Three closely related methyl­ated hydrazine carbamates show different hydrogen-bonding patterns, although they all result in chains.

## Chemical context   

As part of our ongoing studies of hydrazine carbamates derived from l-serine with possible anti-tubercular activity (Pinheiro *et al.*, 2011 ▸), we now describe the syntheses and structures of three methyl­ated derivatives, *viz*: benzyl (*E*)-3-hy­droxy-1-[2-(4-cyano­benzyl­dene)-1-methyl­hydrazin­yl]-1-oxo­propan-2-ylcarbamate (I), *tert*-butyl (*E*)-3-hy­droxy-1-[2-(4-cyano­benzyl­idene)-1-methyl­hydrazin­yl]-1-oxopropan-2-ylcarbamate (II) and *tert*-butyl (*E*)-3-hy­droxy-1-[2-benzyl­idene-1-methyl­hydrazin­yl]-1-oxopropan-2-ylcarbamate (III), formed by the reaction of the corresponding (*E*)-(*S*)-*R*OCONHCH(CH_2_OH)CONHN=CH-benzene (*R* = *t*-Bu or PhCH_2_) compound (Noguiera *et al.*, 2013 ▸) with potassium carbonate and methyl iodide. 
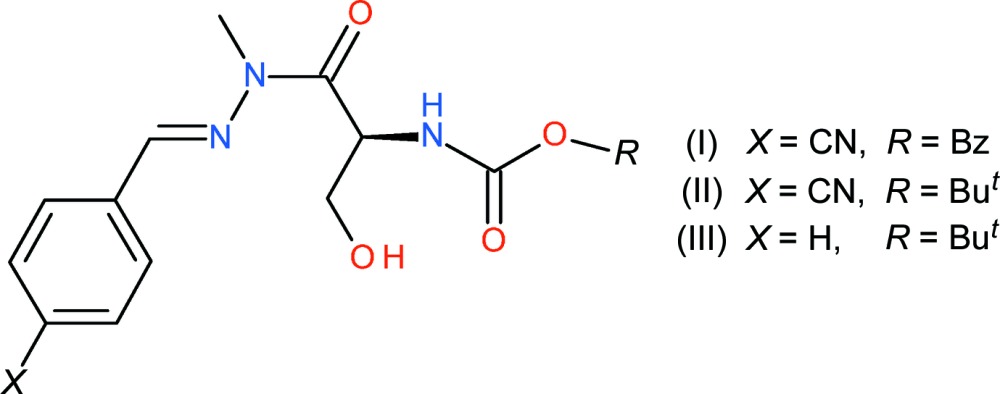


In general, the tertiary butyl compounds form simple methyl­ated products as described here, whereas the benzyl compounds lead to cyclized oxazolidin-2-one products (Noguiera *et al.*, 2013 ▸). However, compound (I) described herein has not cyclized. As described below, compound (III) has undergone an unexpected racemization during the methyl­ation step. The acidity of the α-hydrogen atom in serine derivatives has been variously reported (*e.g*., Blaskovich & Lajoie, 1993 ▸; Kovacs *et al.*, 1984 ▸), and apparently can result in racemization in the presence of even a very weak base such as the carbonate ion. Similar racemizations have been observed in the cyclized oxazolidin-2-one products (Noguiera *et al.*, 2015 ▸).

## Structural commentary   

**Figure 1:**
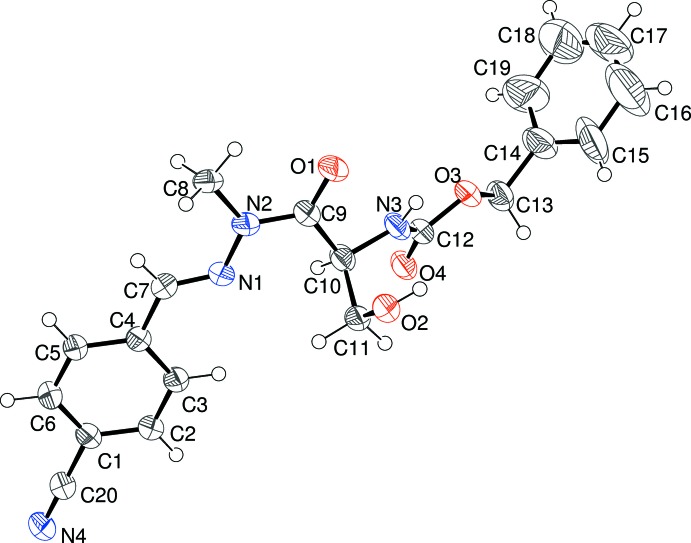
The mol­ecular structure of (I) showing 50% displacement ellipsoids.

The mol­ecular structure of (I) is shown in Fig. 1 ▸, which confirms that methyl­ation has occurred at N2 but no cyclization to an oxazolidin-2-one has occurred (Noguiera *et al.*, 2015 ▸). Compound (I) crystallizes in a chiral space group but its absolute structure was indeterminate in the present experiment and C10 was assumed to have an *S* configuration to match the corresponding atom in the l-serine starting mat­erial. The atoms of the C14 benzene ring show notably larger displacement ellipsoids than the rest of the mol­ecule, but attempts to model this as disorder did not lead to a significant improvement in fit. Atom N2 is statistically planar (bond-angle sum = 360°), which implies *sp* ^2^ hybridization for this atom. The C9—N2 bond length of 1.358 (6)Å is typical of an amide and the N1—N2 bond length of 1.374 (5) is shorter than the reference value of 1.40 Å for a nominal N(*sp* ^2^)—N(*sp* ^2^) single bond. This suggests at least some electronic conjugation over the almost planar C7/N1/N2/C9/O1 grouping (r.m.s. deviation = 0.010 Å): the C1 benzene ring is twisted by 6.1 (2)° with respect to these atoms. The C7—N1—N2—C8 torsion angle of −1.9 (6)° shows that the carbon atoms are almost eclipsed with respect to the N—N bond whereas the C9—C10—C11—O2 torsion angle of −50.9 (5)° indicates a gauche conformation about the C10—C11 bond. The C9—C10—N3—H3*A* torsion angle is 38° and the separation between H2*A* (bonded to O2) and H3*A* is 2.5 Å.

**Figure 2:**
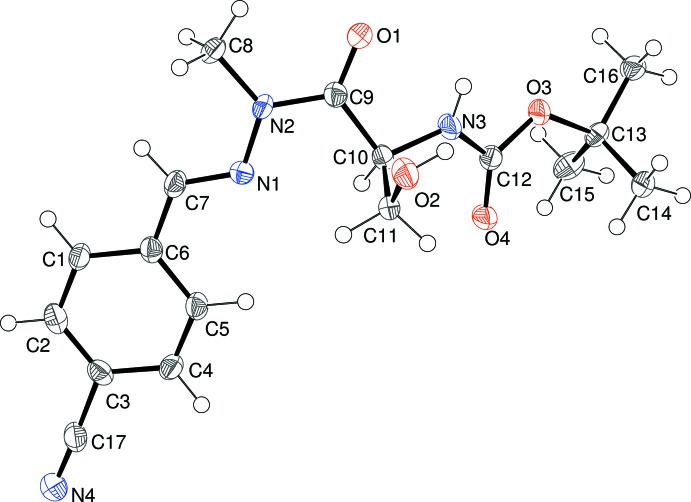
The mol­ecular structure of (II) showing 50% displacement ellipsoids.

The mol­ecular structure of (II) can be seen in Fig. 2 ▸: again the methyl­ation of N2 has occurred as expected. Because the absolute structure was indeterminate, the configuration of C10 (*S*) was assumed to be the same as that of the corresponding atom in the l-serine starting material. In terms of the C7/N1/N2/C9/O1 grouping in (II), the C9—N2 and N1—N2 bond lengths are 1.385 (6) and 1.388 (5) Å, respectively, which are both notably longer than the corresponding bonds in (I), and the r.m.s. deviation from planarity of 0.049 Å for these five atoms is also larger than the corresponding value for (I). The dihedral angle between C7/N1/N2/C9/O1 and the C1-benzene ring in (II) is 10.5 (3)°. The C7—N1—N2—C8 torsion angle is 1.2 (7)° and the C9—C10—C11—O2 torsion angle is −47.4 (6)°, which are similar to the equivalent data for (I). The C9—C10—N3—H3 torsion angle in (II) is 30° and the separation between H2*A* and H3 is 2.7 Å. These values are evidently sufficiently different from the corresponding data for (I) to lead to a different hydrogen-bonding pattern in the crystal (see below).

**Figure 3:**
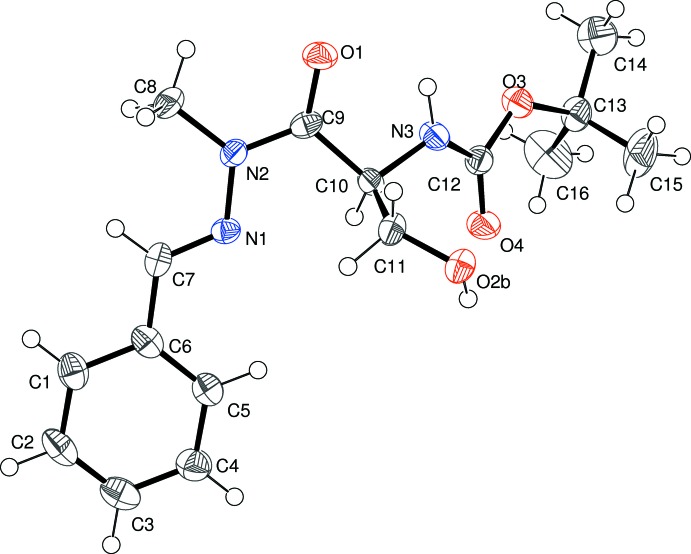
The mol­ecular structure of (III) showing 50% displacement ellipsoids. Only one orientation of the disordered O2—H2 group is shown.

Compound (III), shown in Fig. 3 ▸, crystallizes in a centrosymmetric space group, indicating that racemization of C10 has occurred during the methyl­ation of N2: the C10 atom in the asymmetric unit was arbitrarily assigned an *S* configuration. The O2—H2 hy­droxy group is disordered over two orientations in a 0.802 (7):0.198 (7) ratio. The geometric parameters for (III) are largely consistent with those for (I) and (II): the C7/N1/N2/C9/O1 grouping (r.m.s. deviation = 0.014 Å) subtends a dihedral angle of 1.9 (4)° with the C1–C6 benzene ring and the C9—N2 and N1—N2 bond lengths are 1.358 (5) and 1.381 (4) Å, respectively. The C7—N1—N2—C8 torsion angle is 0.8 (5)° and the C9—C10—C11—O2*A* (major disorder component) torsion angle is −54.9 (4)°. The C9—C10—C11—O2*B* torsion angle for the minor disorder component is −156.7 (8)°, which has a significant role to play in the hydrogen-bonding pattern in the crystal of (III).

## Supra­molecular features   

**Figure 4:**
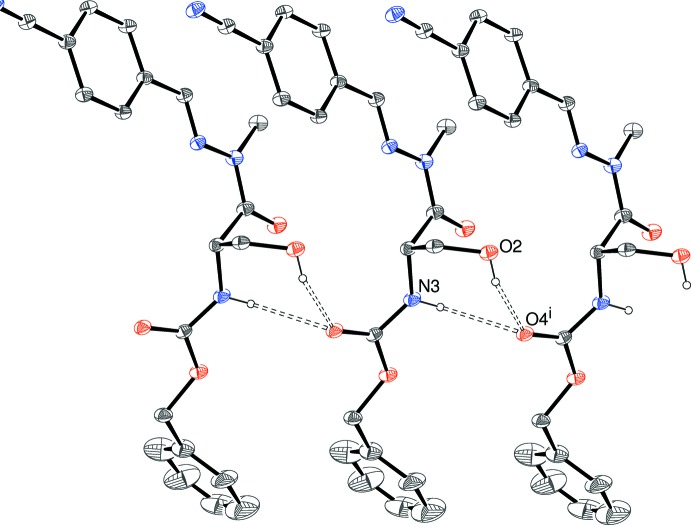
Fragment of a [100] hydrogen-bonded chain in the crystal of (I). Symmetry code: (i) 1 + *x*, *y*, *z*. All C-bound H atoms are omitted for clarity.

In the extended structure of (I), the mol­ecules are linked by short O2—H2*A*⋯O4^i^ (i = 1 + *x*, *y*, *z*) and much longer N3—H3*A*⋯O4^i^ hydrogen bonds (Table 1 ▸, Fig. 4 ▸) to the same acceptor oxygen atom, generating [100] chains, with adjacent mol­ecules related by simple translation in the a-axis direction. An unusual 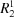
(7) loop arises from these hydrogen bonds; alternately, this could be described as combined *C*(7) O—H⋯O and *C*(4) N—H⋯O chains. A pair of weak C—H⋯π inter­actions are also observed but there is no aromatic π–π stacking (shortest centroid–centroid separation > 4.7 Å).

**Figure 5:**
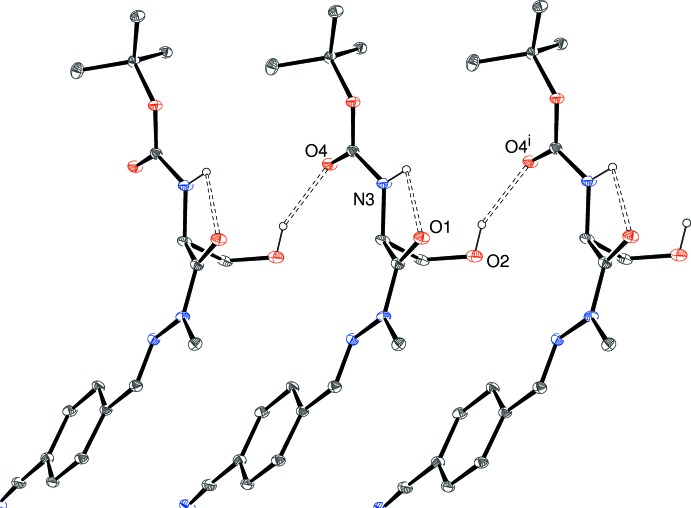
Fragment of a [100] hydrogen-bonded chain in the crystal of (II). Symmetry code: (i) 1 + *x*, *y*, *z*. All C-bound H atoms are omitted for clarity.

The extended structure of (II) also features [100] chains (Fig. 5 ▸) with adjacent mol­ecules related by translation, but in this case the mol­ecules are only linked by *C*(7) O2—H2*A*⋯O4^i^ (i = 1 + *x*, *y*, *z*) hydrogen bonds (Table 2 ▸) with almost the same local geometry as seen in (I). The N3—H3 grouping in (II) is twisted far enough away from O4^i^ to not form an inter­molecular hydrogen bond (H3⋯O4^i^ = 3.2 Å), but instead forms an intra­molecular link to O1. A very long inter­molecular C—H⋯N inter­action is observed but there is no π–π stacking in (II), as the shortest centroid–centroid separation is greater than 5.3 Å.

**Figure 6:**
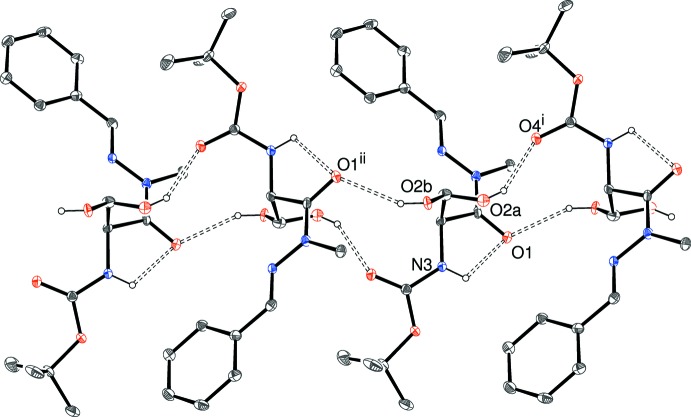
Fragment of an [010] hydrogen-bonded chain in the crystal of (III). Both disorder components of the OH group are shown. Symmetry codes: (i) 1 − *x*, *y* − 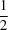, 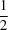 − *z*; (ii) 1 − *x*, *y* + 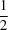, 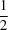 − *z*. All C-bound H atoms are omitted for clarity.

The packing in the centrosymmetric structure of (III) leads to [010] chains (Fig. 6 ▸) with adjacent mol­ecules related by the 2_1_ screw axis, so that the C1-benzene ring is ‘flipped’ from one side of the chain to the other in adjacent mol­ecules. As noted above, the hydroxyl group is disordered over two orientations. The hydrogen bond from the major orientation of O2*A*—H2*A* is still a bond to O4^i^ (Table 3 ▸), where i = 1 − *x*, *y* − 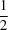, 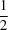 − *z*. The minor disorder component (O2*B*—H2*B*) forms an O—H⋯O hydrogen bond in the opposite chain direction to O1^ii^ (ii = 1 − *x*, *y* + 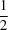, 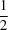 − *z*): O1 also accepts an intra­molecular N—H⋯O hydrogen bond, as seen in (II). Once again, no aromatic π–π stacking is observed in the crystal of (III), as the minimum centroid–centroid separation is greater than 4.6 Å.

## Database survey   

There are no –OCONHCH(CH_2_OH)CON(CH_3_)N=CH– fragments reported in Version 5.36 of the Cambridge Structural Database (Groom & Allen, 2014 ▸) but there are 14 unmethyl­ated –OCONHCH(CH_2_OH)CONHN=CH– groupings with different substituents at each end of the fragment, all of which have been reported by us in the last few years (Howie *et al.*, 2011 ▸ and references therein). All of these materials crystallize in chiral space groups.

## Synthesis and crystallization   

Potassium carbonate (1.76 mmol) was added to a solution of the appropriate (*E*)-(*S*)-*R*OCONHCH(CH_2_OH)CONHN=CH-benzene compound (Noguiera *et al.*, 2013 ▸) in acetone (10 ml) and the reaction mixture was vigorously stirred at room temperature for 5 minutes, before adding methyl iodide (1.80 mmol). The reaction mixture was stirred at 323 K for 24–48 h and the solvent removed by rotary evaporation. The residue was subjected to column chromatography on silica gel, using a chloro­form:methanol (100 → 95%) gradient. The colourless crystals used in the structure determinations were recrystallized from ethanol solution at room temperature. For further details and spectroscopic data, see: Noguiera *et al.* (2013 ▸).

## Refinement   

Crystal data, data collection and structure refinement details are summarized in Table 4 ▸. The crystal of (I) gave a poor diffraction pattern and indexing initially established a large triclinic unit cell [*a* = 9.512 (12), *b* = 13.003 (19), *c* = 22.94 (3) Å, α = 92.93 (2), β = 91.48 (3), γ = 98.13 (3)°, *V* = 2804 (7) Å^3^]. An atomic model could be developed in space group *P*1 with *Z* = 6, but a *PLATON* (Spek, 2009 ▸) symmetry check indicated that the smaller monoclinic cell reported above was more appropriate and the unit cell transformed by the matrix (−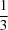 −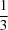 0 / −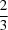 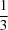 0 / 0 0 −1). It is notable that the aromatic rings of the benzyl groups of all six mol­ecules in the triclinic supercell showed a high degree of thermal motion. The transformation to monoclinic symmetry resulted in a rather low data completion percentage of 92%, but we consider that the refinement is satisfactory and a good geometrical precision results. For each structure, the O- and N-bound H atoms were located in difference maps, repositioned in idealized locations and refined as riding atoms [H1*N* was freely refined in structure (III)]. The C-bound H atoms were placed geometrically (C—H = 0.95–1.00 Å) and refined as riding atoms. The constraint *U*_iso_(H) = 1.2*U*_eq_(carrier) or 1.5*U*_eq_(methyl carrier) was applied in all cases. The H atoms of the hydroxyl groups were allowed to rotate about their C—O bond (*SHELXL* HFIX 83 instruction with O—H = 0.84 Å and C—O—H = 109.5°) to best fit the electron density. The methyl groups were allowed to rotate, but not to tip, to best fit the electron density (AFIX 137 instruction).

## Supplementary Material

Crystal structure: contains datablock(s) I, II, III, global. DOI: 10.1107/S2056989015010440/hg5443sup1.cif

Structure factors: contains datablock(s) I. DOI: 10.1107/S2056989015010440/hg5443Isup2.hkl

Structure factors: contains datablock(s) II. DOI: 10.1107/S2056989015010440/hg5443IIsup3.hkl

Structure factors: contains datablock(s) III. DOI: 10.1107/S2056989015010440/hg5443IIIsup4.hkl

Supporting information file. DOI: 10.1107/S2056989015010440/hg5443Isup5.cml

Supporting information file. DOI: 10.1107/S2056989015010440/hg5443IIsup6.cml

Supporting information file. DOI: 10.1107/S2056989015010440/hg5443IIIsup7.cml

CCDC references: 1404006, 1404005, 1404004

Additional supporting information:  crystallographic information; 3D view; checkCIF report

## Figures and Tables

**Table 1:** Hydrogen-bond geometry (Å, °) for (I) *Cg*2 is the centroid of the C14-C19 ring.

*D*—H⋯*A*	*D*—H	H⋯*A*	*D*⋯*A*	*D*—H⋯*A*
N3—H3*A*⋯O4^i^	0.88	2.40	3.091 (6)	135
O2—H2*A*⋯O4^i^	0.84	2.08	2.873 (5)	158
C16—H16⋯*Cg*2^ii^	0.95	2.78	3.558 (18)	140
C19—H19⋯*Cg*2^iii^	0.95	2.86	3.598 (13)	135

Symmetry codes: (i) 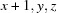; (ii) 
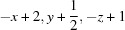
; (iii) 
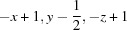
.

**Table 2:** Hydrogen-bond geometry (Å, °) for (II)

*D*—H⋯*A*	*D*—H	H⋯*A*	*D*⋯*A*	*D*—H⋯*A*
N3—H3⋯O1	0.88	2.27	2.620 (6)	104
O2—H2*A*⋯O4^i^	0.84	2.09	2.877 (5)	156
C4—H4⋯N4^ii^	0.95	2.61	3.549 (8)	168

Symmetry codes: (i) 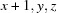; (ii) 
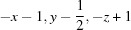
.

**Table 3:** Hydrogen-bond geometry (Å, °) for (III)

*D*—H⋯*A*	*D*—H	H⋯*A*	*D*⋯*A*	*D*—H⋯*A*
N3—H1*N*⋯O1	0.88 (4)	2.11 (4)	2.623 (4)	116 (3)
O2*A*—H2*A*⋯O4^i^	0.84	2.09	2.852 (4)	150
O2*B*—H2*B*⋯O1^ii^	0.84	2.18	2.966 (13)	156
C7—H7⋯O2*A* ^iii^	0.95	2.45	3.229 (5)	140

Symmetry codes: (i) 
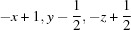
; (ii) 
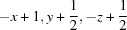
; (iii) 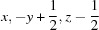.

**Table 4:** Experimental details

	(I)	(II)	(III)
Crystal data			
Chemical formula	C_20_H_20_N_4_O_4_	C_17_H_22_N_4_O_4_	C_16_H_23_N_3_O_4_
*M*_r_	380.40	346.39	321.37
Crystal system, space group	Monoclinic, *P*2_1_	Monoclinic, *P*2_1_	Monoclinic, *P*2_1_/*c*
Temperature (K)	100	100	100
*a*, *b*, *c* (Å)	4.995 (6), 8.172 (8), 22.94 (3)	5.348 (3), 7.883 (5), 20.903 (14)	10.454 (7), 10.571 (7), 15.664 (11)
β (°)	93.48 (3)	92.763 (1)	101.172 (12)
*V* (Å^3^)	934.7 (19)	880.2 (10)	1698 (2)
*Z*	2	2	4
Radiation type	Mo *K*α	Mo *K*α	Mo *K*α
μ (mm^−1^)	0.10	0.10	0.09
Crystal size (mm)	0.14 × 0.03 × 0.01	0.08 × 0.08 × 0.02	0.16 × 0.05 × 0.01

Data collection			
Diffractometer	Rigaku Mercury CCD	Rigaku Mercury CCD	Rigaku Mercury CCD
No. of measured, independent and observed [*I* > 2σ(*I*)] reflections	13928, 4691, 3270	3672, 2483, 2143	8546, 3319, 2716
*R*_int_	0.070	0.023	0.048
(sin θ/λ)_max_ (Å^−1^)	0.734	0.617	0.617

Refinement			
*R*[*F* ^2^ > 2σ(*F* ^2^)], *wR*(*F* ^2^), *S*	0.095, 0.278, 1.10	0.057, 0.194, 1.13	0.104, 0.197, 1.23
No. of reflections	4691	2483	3319
No. of parameters	255	231	220
No. of restraints	1	1	0
H-atom treatment	H-atom parameters constrained	H-atom parameters constrained	H atoms treated by a mixture of independent and constrained refinement
Δρ_max_, Δρ_min_ (e Å^−3^)	0.39, −0.35	0.31, −0.36	0.44, −0.26

Computer programs: *CrystalClear* (Rigaku, 2012 ▸), *SHELXS97* and *SHELXL97* (Sheldrick, 2008 ▸), *ORTEP-3 for Windows* (Farrugia, 2012 ▸) and *publCIF* (Westrip, 2010 ▸).

## supplementary crystallographic information

### (I) Benzyl N-{(E)-1-[2-(4-cyanobenzylidene)-1-methylhydrazinyl]-3-hydroxy-1-oxopropan-2-yl}carbamate Crystal data

**Table:**

C_20_H_20_N_4_O_4_	*F*(000) = 400
*M**_r_* = 380.40	*D*_x_ = 1.352 Mg m^−^^3^
Monoclinic, *P*2_1_	Mo *K*α radiation, λ = 0.71073 Å
Hall symbol: P 2yb	Cell parameters from 5405 reflections
*a* = 4.995 (6) Å	θ = 2.2–31.3°
*b* = 8.172 (8) Å	µ = 0.10 mm^−^^1^
*c* = 22.94 (3) Å	*T* = 100 K
β = 93.48 (3)°	Chip, colourless
*V* = 934.7 (19) Å^3^	0.14 × 0.03 × 0.01 mm
*Z* = 2	

### (I) Benzyl N-{(E)-1-[2-(4-cyanobenzylidene)-1-methylhydrazinyl]-3-hydroxy-1-oxopropan-2-yl}carbamate Data collection

**Table:**

Rigaku Mercury CCD diffractometer	3270 reflections with *I* > 2σ(*I*)
Radiation source: fine-focus sealed tube	*R*_int_ = 0.070
Graphite monochromator	θ_max_ = 31.5°, θ_min_ = 2.7°
ω scans	*h* = −7→7
13928 measured reflections	*k* = −11→7
4691 independent reflections	*l* = −32→33

### (I) Benzyl N-{(E)-1-[2-(4-cyanobenzylidene)-1-methylhydrazinyl]-3-hydroxy-1-oxopropan-2-yl}carbamate Refinement

**Table:**

Refinement on *F*^2^	Secondary atom site location: difference Fourier map
Least-squares matrix: full	Hydrogen site location: inferred from neighbouring sites
*R*[*F*^2^ > 2σ(*F*^2^)] = 0.095	H-atom parameters constrained
*wR*(*F*^2^) = 0.278	*w* = 1/[σ^2^(*F*_o_^2^) + (0.1373*P*)^2^ + 0.3363*P*] where *P* = (*F*_o_^2^ + 2*F*_c_^2^)/3
*S* = 1.10	(Δ/σ)_max_ = 0.005
4691 reflections	Δρ_max_ = 0.39 e Å^−^^3^
255 parameters	Δρ_min_ = −0.35 e Å^−^^3^
1 restraint	Extinction correction: *SHELXL*, Fc^*^=kFc[1+0.001xFc^2^λ^3^/sin(2θ)]^-1/4^
Primary atom site location: structure-invariant direct methods	Extinction coefficient: 0.079 (13)

### (I) Benzyl N-{(E)-1-[2-(4-cyanobenzylidene)-1-methylhydrazinyl]-3-hydroxy-1-oxopropan-2-yl}carbamate Special details

**Table:**

Geometry. All e.s.d.'s (except the e.s.d. in the dihedral angle between two l.s. planes) are estimated using the full covariance matrix. The cell e.s.d.'s are taken into account individually in the estimation of e.s.d.'s in distances, angles and torsion angles; correlations between e.s.d.'s in cell parameters are only used when they are defined by crystal symmetry. An approximate (isotropic) treatment of cell e.s.d.'s is used for estimating e.s.d.'s involving l.s. planes.
Refinement. Refinement of *F*^2^ against ALL reflections. The weighted *R*-factor *wR* and goodness of fit *S* are based on *F*^2^, conventional *R*-factors *R* are based on *F*, with *F* set to zero for negative *F*^2^. The threshold expression of *F*^2^ > σ(*F*^2^) is used only for calculating *R*-factors(gt) *etc*. and is not relevant to the choice of reflections for refinement. *R*-factors based on *F*^2^ are statistically about twice as large as those based on *F*, and *R*- factors based on ALL data will be even larger.

### (I) Benzyl N-{(E)-1-[2-(4-cyanobenzylidene)-1-methylhydrazinyl]-3-hydroxy-1-oxopropan-2-yl}carbamate Fractional atomic coordinates and isotropic or equivalent isotropic displacement parameters (Å^2^)

**Table:**

	*x*	*y*	*z*	*U*_iso_*/*U*_eq_	
C1	−0.1557 (8)	−0.2306 (6)	0.04534 (16)	0.0387 (8)	
C2	−0.0584 (9)	−0.0833 (5)	0.06903 (16)	0.0389 (9)	
H2	−0.1380	0.0174	0.0567	0.047*	
C3	0.1564 (8)	−0.0847 (5)	0.11093 (16)	0.0371 (8)	
H3	0.2225	0.0153	0.1273	0.044*	
C4	0.2746 (8)	−0.2330 (5)	0.12891 (15)	0.0358 (8)	
C5	0.1737 (9)	−0.3795 (5)	0.10501 (16)	0.0393 (9)	
H5	0.2541	−0.4802	0.1171	0.047*	
C6	−0.0422 (9)	−0.3803 (6)	0.06378 (18)	0.0404 (9)	
H6	−0.1116	−0.4806	0.0483	0.048*	
C7	0.5009 (8)	−0.2375 (6)	0.17309 (16)	0.0378 (8)	
H7	0.5872	−0.3388	0.1820	0.045*	
C8	0.9329 (10)	−0.2648 (6)	0.25743 (18)	0.0443 (10)	
H8A	1.0041	−0.3156	0.2229	0.066*	
H8B	0.8063	−0.3400	0.2745	0.066*	
H8C	1.0808	−0.2409	0.2862	0.066*	
C9	0.8664 (10)	0.0289 (6)	0.26783 (18)	0.0416 (9)	
C10	0.7063 (9)	0.1831 (5)	0.24991 (18)	0.0388 (9)	
H10	0.5107	0.1563	0.2457	0.047*	
C11	0.7983 (9)	0.2508 (5)	0.19144 (16)	0.0396 (9)	
H11A	0.7380	0.1753	0.1595	0.048*	
H11B	0.7112	0.3580	0.1835	0.048*	
C12	0.5656 (8)	0.4037 (6)	0.31560 (16)	0.0379 (8)	
C13	0.4928 (10)	0.6097 (6)	0.3865 (2)	0.0477 (11)	
H13A	0.3119	0.5643	0.3916	0.057*	
H13B	0.4753	0.7055	0.3602	0.057*	
C14	0.6244 (14)	0.6577 (9)	0.4441 (2)	0.0688 (17)	
C15	0.8449 (18)	0.7581 (12)	0.4483 (4)	0.105 (3)	
H15	0.9131	0.8020	0.4139	0.126*	
C16	0.970 (3)	0.796 (2)	0.5025 (7)	0.163 (5)	
H16	1.1220	0.8664	0.5053	0.195*	
C17	0.871 (3)	0.7322 (17)	0.5505 (4)	0.141 (5)	
H17	0.9569	0.7613	0.5872	0.169*	
C18	0.671 (3)	0.6355 (18)	0.5509 (4)	0.149 (5)	
H18	0.6174	0.5882	0.5862	0.179*	
C19	0.531 (3)	0.6011 (13)	0.4962 (3)	0.125 (4)	
H19	0.3715	0.5381	0.4954	0.150*	
C20	−0.3731 (8)	−0.2306 (6)	0.00082 (17)	0.0411 (9)	
N1	0.5835 (7)	−0.1082 (5)	0.19964 (14)	0.0360 (7)	
N2	0.7956 (8)	−0.1136 (5)	0.24061 (15)	0.0407 (8)	
N3	0.7554 (7)	0.2999 (5)	0.29708 (15)	0.0422 (8)	
H3A	0.9170	0.3037	0.3146	0.051*	
N4	−0.5474 (8)	−0.2307 (6)	−0.03581 (15)	0.0480 (9)	
O1	1.0542 (7)	0.0348 (4)	0.30583 (13)	0.0503 (9)	
O2	1.0784 (6)	0.2702 (4)	0.19126 (12)	0.0445 (7)	
H2A	1.1303	0.3363	0.2176	0.067*	
O3	0.6668 (7)	0.4855 (4)	0.36255 (12)	0.0440 (8)	
O4	0.3405 (6)	0.4203 (4)	0.29257 (13)	0.0443 (7)	

### (I) Benzyl N-{(E)-1-[2-(4-cyanobenzylidene)-1-methylhydrazinyl]-3-hydroxy-1-oxopropan-2-yl}carbamate Atomic displacement parameters (Å^2^)

**Table:**

	*U*^11^	*U*^22^	*U*^33^	*U*^12^	*U*^13^	*U*^23^
C1	0.046 (2)	0.038 (2)	0.0309 (16)	−0.0001 (19)	−0.0025 (14)	−0.0043 (17)
C2	0.048 (2)	0.035 (2)	0.0324 (17)	−0.0014 (19)	−0.0040 (15)	0.0005 (16)
C3	0.044 (2)	0.032 (2)	0.0344 (17)	0.0029 (18)	−0.0044 (15)	−0.0008 (15)
C4	0.043 (2)	0.0313 (19)	0.0324 (16)	0.0012 (18)	−0.0016 (14)	−0.0003 (16)
C5	0.053 (2)	0.031 (2)	0.0333 (18)	−0.0002 (18)	−0.0037 (16)	−0.0020 (15)
C6	0.049 (2)	0.035 (2)	0.0365 (19)	−0.0059 (18)	−0.0001 (16)	−0.0050 (16)
C7	0.044 (2)	0.0329 (19)	0.0360 (17)	−0.0007 (18)	−0.0012 (15)	0.0032 (16)
C8	0.060 (3)	0.040 (2)	0.0316 (17)	0.005 (2)	−0.0073 (17)	0.0056 (16)
C9	0.050 (2)	0.039 (2)	0.0346 (18)	−0.0028 (19)	−0.0080 (16)	−0.0021 (17)
C10	0.039 (2)	0.036 (2)	0.0397 (19)	0.0014 (17)	−0.0100 (16)	−0.0058 (16)
C11	0.052 (2)	0.030 (2)	0.0355 (18)	0.0038 (18)	−0.0102 (16)	−0.0012 (15)
C12	0.043 (2)	0.037 (2)	0.0324 (17)	−0.0061 (18)	−0.0066 (15)	−0.0023 (16)
C13	0.057 (3)	0.044 (3)	0.042 (2)	0.000 (2)	−0.0026 (19)	−0.0129 (19)
C14	0.092 (4)	0.071 (4)	0.042 (2)	0.003 (3)	−0.009 (3)	−0.016 (3)
C15	0.116 (6)	0.087 (6)	0.107 (6)	−0.015 (5)	−0.035 (5)	−0.048 (5)
C16	0.189 (11)	0.144 (11)	0.145 (9)	−0.013 (10)	−0.080 (9)	−0.057 (9)
C17	0.232 (15)	0.110 (9)	0.075 (6)	0.032 (9)	−0.048 (8)	−0.044 (6)
C18	0.242 (16)	0.140 (11)	0.066 (5)	−0.048 (11)	0.006 (7)	−0.020 (6)
C19	0.207 (11)	0.113 (8)	0.054 (4)	0.005 (8)	0.005 (5)	−0.011 (5)
C20	0.046 (2)	0.038 (2)	0.0390 (18)	−0.0037 (19)	−0.0011 (16)	−0.0022 (18)
N1	0.0400 (17)	0.0363 (18)	0.0308 (14)	−0.0027 (15)	−0.0057 (12)	0.0015 (13)
N2	0.050 (2)	0.0362 (19)	0.0343 (15)	0.0043 (16)	−0.0108 (14)	0.0038 (14)
N3	0.0452 (18)	0.042 (2)	0.0372 (16)	−0.0023 (16)	−0.0142 (13)	−0.0077 (14)
N4	0.053 (2)	0.047 (2)	0.0423 (18)	−0.0066 (19)	−0.0120 (15)	−0.0048 (18)
O1	0.058 (2)	0.0464 (19)	0.0429 (16)	0.0028 (16)	−0.0232 (14)	0.0009 (14)
O2	0.0532 (17)	0.0387 (16)	0.0403 (14)	−0.0021 (14)	−0.0072 (12)	−0.0024 (13)
O3	0.0546 (19)	0.0439 (17)	0.0321 (13)	−0.0040 (14)	−0.0090 (12)	−0.0074 (12)
O4	0.0466 (16)	0.0429 (18)	0.0421 (15)	−0.0021 (14)	−0.0093 (12)	−0.0093 (13)

### (I) Benzyl N-{(E)-1-[2-(4-cyanobenzylidene)-1-methylhydrazinyl]-3-hydroxy-1-oxopropan-2-yl}carbamate Geometric parameters (Å, º)

**Table:**

C1—C2	1.395 (6)	C11—H11A	0.9900
C1—C6	1.404 (7)	C11—H11B	0.9900
C1—C20	1.445 (5)	C12—O4	1.221 (5)
C2—C3	1.397 (6)	C12—O3	1.340 (5)
C2—H2	0.9500	C12—N3	1.359 (6)
C3—C4	1.399 (6)	C13—O3	1.465 (6)
C3—H3	0.9500	C13—C14	1.492 (7)
C4—C5	1.398 (6)	C13—H13A	0.9900
C4—C7	1.472 (5)	C13—H13B	0.9900
C5—C6	1.391 (6)	C14—C15	1.372 (11)
C5—H5	0.9500	C14—C19	1.386 (11)
C6—H6	0.9500	C15—C16	1.391 (12)
C7—N1	1.276 (6)	C15—H15	0.9500
C7—H7	0.9500	C16—C17	1.34 (2)
C8—N2	1.454 (6)	C16—H16	0.9500
C8—H8A	0.9800	C17—C18	1.274 (17)
C8—H8B	0.9800	C17—H17	0.9500
C8—H8C	0.9800	C18—C19	1.428 (16)
C9—O1	1.242 (5)	C18—H18	0.9500
C9—N2	1.358 (6)	C19—H19	0.9500
C9—C10	1.535 (6)	C20—N4	1.173 (5)
C10—N3	1.453 (5)	N1—N2	1.374 (5)
C10—C11	1.546 (6)	N3—H3A	0.8800
C10—H10	1.0000	O2—H2A	0.8400
C11—O2	1.409 (6)		
			
C2—C1—C6	120.6 (3)	C10—C11—H11B	109.0
C2—C1—C20	120.3 (4)	H11A—C11—H11B	107.8
C6—C1—C20	119.0 (4)	O4—C12—O3	125.8 (4)
C1—C2—C3	119.7 (4)	O4—C12—N3	125.2 (4)
C1—C2—H2	120.1	O3—C12—N3	109.0 (3)
C3—C2—H2	120.1	O3—C13—C14	106.0 (4)
C2—C3—C4	120.2 (4)	O3—C13—H13A	110.5
C2—C3—H3	119.9	C14—C13—H13A	110.5
C4—C3—H3	119.9	O3—C13—H13B	110.5
C5—C4—C3	119.4 (3)	C14—C13—H13B	110.5
C5—C4—C7	119.5 (4)	H13A—C13—H13B	108.7
C3—C4—C7	121.2 (4)	C15—C14—C19	116.6 (8)
C6—C5—C4	121.2 (4)	C15—C14—C13	121.9 (6)
C6—C5—H5	119.4	C19—C14—C13	121.5 (8)
C4—C5—H5	119.4	C14—C15—C16	120.8 (11)
C5—C6—C1	118.9 (4)	C14—C15—H15	119.6
C5—C6—H6	120.6	C16—C15—H15	119.6
C1—C6—H6	120.6	C17—C16—C15	118.6 (13)
N1—C7—C4	121.3 (4)	C17—C16—H16	120.7
N1—C7—H7	119.3	C15—C16—H16	120.7
C4—C7—H7	119.3	C18—C17—C16	125.1 (10)
N2—C8—H8A	109.5	C18—C17—H17	117.4
N2—C8—H8B	109.5	C16—C17—H17	117.4
H8A—C8—H8B	109.5	C17—C18—C19	117.2 (11)
N2—C8—H8C	109.5	C17—C18—H18	121.4
H8A—C8—H8C	109.5	C19—C18—H18	121.4
H8B—C8—H8C	109.5	C14—C19—C18	121.4 (12)
O1—C9—N2	121.3 (4)	C14—C19—H19	119.3
O1—C9—C10	121.0 (4)	C18—C19—H19	119.3
N2—C9—C10	117.6 (3)	N4—C20—C1	179.2 (4)
N3—C10—C9	106.2 (3)	C7—N1—N2	120.9 (4)
N3—C10—C11	111.4 (4)	C9—N2—N1	117.1 (3)
C9—C10—C11	110.4 (4)	C9—N2—C8	120.1 (3)
N3—C10—H10	109.6	N1—N2—C8	122.7 (3)
C9—C10—H10	109.6	C12—N3—C10	123.6 (3)
C11—C10—H10	109.6	C12—N3—H3A	118.2
O2—C11—C10	113.0 (3)	C10—N3—H3A	118.2
O2—C11—H11A	109.0	C11—O2—H2A	109.5
C10—C11—H11A	109.0	C12—O3—C13	116.3 (4)
O2—C11—H11B	109.0		
			
C6—C1—C2—C3	0.8 (6)	C14—C15—C16—C17	0 (2)
C20—C1—C2—C3	−178.3 (4)	C15—C16—C17—C18	1 (2)
C1—C2—C3—C4	0.3 (6)	C16—C17—C18—C19	−4 (2)
C2—C3—C4—C5	−0.7 (6)	C15—C14—C19—C18	−4.7 (15)
C2—C3—C4—C7	−179.9 (3)	C13—C14—C19—C18	174.5 (10)
C3—C4—C5—C6	−0.1 (6)	C17—C18—C19—C14	6 (2)
C7—C4—C5—C6	179.1 (3)	C2—C1—C20—N4	94 (37)
C4—C5—C6—C1	1.2 (6)	C6—C1—C20—N4	−85 (37)
C2—C1—C6—C5	−1.6 (6)	C4—C7—N1—N2	−179.7 (3)
C20—C1—C6—C5	177.6 (4)	O1—C9—N2—N1	179.3 (4)
C5—C4—C7—N1	−173.8 (4)	C10—C9—N2—N1	−0.8 (6)
C3—C4—C7—N1	5.4 (6)	O1—C9—N2—C8	2.6 (7)
O1—C9—C10—N3	−19.1 (6)	C10—C9—N2—C8	−177.5 (4)
N2—C9—C10—N3	161.0 (4)	C7—N1—N2—C9	−178.4 (4)
O1—C9—C10—C11	101.8 (5)	C7—N1—N2—C8	−1.8 (6)
N2—C9—C10—C11	−78.1 (5)	O4—C12—N3—C10	−5.8 (7)
N3—C10—C11—O2	66.8 (5)	O3—C12—N3—C10	174.9 (4)
C9—C10—C11—O2	−50.9 (5)	C9—C10—N3—C12	−142.7 (4)
O3—C13—C14—C15	76.1 (8)	C11—C10—N3—C12	97.1 (5)
O3—C13—C14—C19	−103.1 (8)	O4—C12—O3—C13	−2.4 (6)
C19—C14—C15—C16	1.5 (15)	N3—C12—O3—C13	176.9 (4)
C13—C14—C15—C16	−177.6 (10)	C14—C13—O3—C12	168.8 (4)

### (I) Benzyl N-{(E)-1-[2-(4-cyanobenzylidene)-1-methylhydrazinyl]-3-hydroxy-1-oxopropan-2-yl}carbamate Hydrogen-bond geometry (Å, º)

Cg2 is the centroid of the C14-C19 ring.

**Table:**

*D*—H···*A*	*D*—H	H···*A*	*D*···*A*	*D*—H···*A*
N3—H3*A*···O4^i^	0.88	2.40	3.091 (6)	135
O2—H2*A*···O4^i^	0.84	2.08	2.873 (5)	158
C16—H16···*Cg*2^ii^	0.95	2.78	3.558 (18)	140
C19—H19···*Cg*2^iii^	0.95	2.86	3.598 (13)	135

Symmetry codes: (i) *x*+1, *y*, *z*; (ii) −*x*+2, *y*+1/2, −*z*+1; (iii) −*x*+1, *y*−1/2, −*z*+1.

### (II) tert-Butyl N-{(E)-1-[2-(4-cyanobenzylidene)-1-methylhydrazinyl]-3-hydroxy-1-oxopropan-2-yl}carbamate Crystal data

**Table:**

C_17_H_22_N_4_O_4_	*F*(000) = 368
*M**_r_* = 346.39	*D*_x_ = 1.307 Mg m^−^^3^
Monoclinic, *P*2_1_	Mo *K*α radiation, λ = 0.71073 Å
Hall symbol: P 2yb	Cell parameters from 2232 reflections
*a* = 5.348 (3) Å	θ = 2.6–31.2°
*b* = 7.883 (5) Å	µ = 0.10 mm^−^^1^
*c* = 20.903 (14) Å	*T* = 100 K
β = 92.763 (1)°	Slab, colourless
*V* = 880.2 (10) Å^3^	0.08 × 0.08 × 0.02 mm
*Z* = 2	

### (II) tert-Butyl N-{(E)-1-[2-(4-cyanobenzylidene)-1-methylhydrazinyl]-3-hydroxy-1-oxopropan-2-yl}carbamate Data collection

**Table:**

Rigaku Mercury CCD diffractometer	2143 reflections with *I* > 2σ(*I*)
Radiation source: fine-focus sealed tube	*R*_int_ = 0.023
Graphite monochromator	θ_max_ = 26.0°, θ_min_ = 2.9°
ω scans	*h* = −6→5
3672 measured reflections	*k* = −9→7
2483 independent reflections	*l* = −22→25

### (II) tert-Butyl N-{(E)-1-[2-(4-cyanobenzylidene)-1-methylhydrazinyl]-3-hydroxy-1-oxopropan-2-yl}carbamate Refinement

**Table:**

Refinement on *F*^2^	Secondary atom site location: difference Fourier map
Least-squares matrix: full	Hydrogen site location: inferred from neighbouring sites
*R*[*F*^2^ > 2σ(*F*^2^)] = 0.057	H-atom parameters constrained
*wR*(*F*^2^) = 0.194	*w* = 1/[σ^2^(*F*_o_^2^) + (0.0993*P*)^2^ + 0.4415*P*] where *P* = (*F*_o_^2^ + 2*F*_c_^2^)/3
*S* = 1.13	(Δ/σ)_max_ < 0.001
2483 reflections	Δρ_max_ = 0.31 e Å^−^^3^
231 parameters	Δρ_min_ = −0.35 e Å^−^^3^
1 restraint	Extinction correction: *SHELXL*, Fc^*^=kFc[1+0.001xFc^2^λ^3^/sin(2θ)]^-1/4^
Primary atom site location: structure-invariant direct methods	Extinction coefficient: 0.027 (8)

### (II) tert-Butyl N-{(E)-1-[2-(4-cyanobenzylidene)-1-methylhydrazinyl]-3-hydroxy-1-oxopropan-2-yl}carbamate Special details

**Table:**

Geometry. All e.s.d.'s (except the e.s.d. in the dihedral angle between two l.s. planes) are estimated using the full covariance matrix. The cell e.s.d.'s are taken into account individually in the estimation of e.s.d.'s in distances, angles and torsion angles; correlations between e.s.d.'s in cell parameters are only used when they are defined by crystal symmetry. An approximate (isotropic) treatment of cell e.s.d.'s is used for estimating e.s.d.'s involving l.s. planes.
Refinement. Refinement of *F*^2^ against ALL reflections. The weighted *R*-factor *wR* and goodness of fit *S* are based on *F*^2^, conventional *R*-factors *R* are based on *F*, with *F* set to zero for negative *F*^2^. The threshold expression of *F*^2^ > σ(*F*^2^) is used only for calculating *R*-factors(gt) *etc*. and is not relevant to the choice of reflections for refinement. *R*-factors based on *F*^2^ are statistically about twice as large as those based on *F*, and *R*- factors based on ALL data will be even larger.

### (II) tert-Butyl N-{(E)-1-[2-(4-cyanobenzylidene)-1-methylhydrazinyl]-3-hydroxy-1-oxopropan-2-yl}carbamate Fractional atomic coordinates and isotropic or equivalent isotropic displacement parameters (Å^2^)

**Table:**

	*x*	*y*	*z*	*U*_iso_*/*U*_eq_	
C1	0.1123 (9)	0.5292 (7)	0.3916 (2)	0.0253 (11)	
H1	0.1851	0.6345	0.3806	0.030*	
C2	−0.0770 (9)	0.5247 (7)	0.4346 (2)	0.0283 (12)	
H2	−0.1325	0.6272	0.4532	0.034*	
C3	−0.1848 (9)	0.3741 (8)	0.4505 (2)	0.0268 (11)	
C4	−0.1071 (10)	0.2210 (7)	0.4233 (2)	0.0273 (12)	
H4	−0.1829	0.1170	0.4347	0.033*	
C5	0.0802 (9)	0.2224 (7)	0.3799 (2)	0.0260 (12)	
H5	0.1300	0.1194	0.3606	0.031*	
C6	0.1972 (9)	0.3760 (7)	0.3641 (2)	0.0235 (10)	
C7	0.3919 (9)	0.3849 (7)	0.3194 (2)	0.0249 (10)	
H7	0.4750	0.4895	0.3127	0.030*	
C8	0.7748 (9)	0.4254 (6)	0.2344 (2)	0.0254 (11)	
H8A	0.8529	0.4655	0.2750	0.038*	
H8B	0.6564	0.5110	0.2174	0.038*	
H8C	0.9043	0.4061	0.2036	0.038*	
C9	0.6871 (9)	0.1258 (7)	0.2084 (2)	0.0242 (11)	
C10	0.5475 (10)	−0.0357 (6)	0.2249 (2)	0.0264 (12)	
H10	0.3697	−0.0078	0.2334	0.032*	
C11	0.6754 (9)	−0.1158 (7)	0.2847 (2)	0.0279 (11)	
H11A	0.6336	−0.0478	0.3226	0.034*	
H11B	0.6061	−0.2311	0.2903	0.034*	
C12	0.3719 (9)	−0.2554 (7)	0.1527 (2)	0.0234 (11)	
C13	0.2467 (9)	−0.4533 (7)	0.0650 (2)	0.0234 (11)	
C14	0.3177 (9)	−0.6125 (7)	0.1010 (2)	0.0264 (11)	
H14A	0.2701	−0.6018	0.1456	0.040*	
H14B	0.4989	−0.6300	0.1001	0.040*	
H14C	0.2303	−0.7095	0.0810	0.040*	
C15	−0.0334 (9)	−0.4161 (8)	0.0670 (3)	0.0333 (13)	
H15A	−0.0711	−0.3082	0.0453	0.050*	
H15B	−0.0803	−0.4087	0.1116	0.050*	
H15C	−0.1285	−0.5074	0.0453	0.050*	
C16	0.3205 (9)	−0.4594 (7)	−0.0040 (2)	0.0278 (12)	
H16A	0.2902	−0.3484	−0.0241	0.042*	
H16B	0.2205	−0.5458	−0.0272	0.042*	
H16C	0.4985	−0.4880	−0.0054	0.042*	
C17	−0.3795 (10)	0.3679 (8)	0.4971 (2)	0.0306 (12)	
N1	0.4545 (7)	0.2521 (5)	0.28820 (18)	0.0224 (9)	
N2	0.6425 (8)	0.2680 (5)	0.24509 (19)	0.0233 (9)	
N3	0.5555 (8)	−0.1424 (6)	0.16859 (19)	0.0280 (10)	
H3	0.6837	−0.1335	0.1439	0.034*	
N4	−0.5229 (9)	0.3672 (7)	0.5357 (2)	0.0356 (11)	
O1	0.8318 (6)	0.1316 (5)	0.16515 (16)	0.0292 (9)	
O2	0.9367 (7)	−0.1279 (5)	0.28307 (16)	0.0339 (9)	
H2A	0.9742	−0.1874	0.2516	0.041*	
O3	0.3944 (6)	−0.3085 (5)	0.09164 (16)	0.0256 (8)	
O4	0.2146 (6)	−0.3015 (5)	0.18845 (16)	0.0292 (9)	

### (II) tert-Butyl N-{(E)-1-[2-(4-cyanobenzylidene)-1-methylhydrazinyl]-3-hydroxy-1-oxopropan-2-yl}carbamate Atomic displacement parameters (Å^2^)

**Table:**

	*U*^11^	*U*^22^	*U*^33^	*U*^12^	*U*^13^	*U*^23^
C1	0.032 (3)	0.017 (3)	0.027 (2)	−0.001 (2)	0.000 (2)	−0.002 (2)
C2	0.035 (3)	0.027 (3)	0.023 (2)	0.009 (3)	−0.004 (2)	−0.005 (2)
C3	0.031 (2)	0.031 (3)	0.018 (2)	0.003 (3)	0.0011 (18)	−0.002 (2)
C4	0.036 (3)	0.020 (3)	0.026 (3)	−0.006 (2)	0.000 (2)	0.000 (2)
C5	0.030 (2)	0.020 (3)	0.028 (3)	0.005 (2)	0.002 (2)	−0.002 (2)
C6	0.030 (2)	0.021 (3)	0.019 (2)	0.004 (2)	−0.0026 (18)	−0.001 (2)
C7	0.033 (2)	0.016 (2)	0.026 (2)	0.003 (2)	−0.0020 (19)	0.001 (2)
C8	0.029 (3)	0.018 (3)	0.029 (3)	−0.005 (2)	0.000 (2)	0.002 (2)
C9	0.029 (3)	0.021 (3)	0.022 (2)	0.003 (2)	0.0025 (19)	0.000 (2)
C10	0.031 (3)	0.022 (3)	0.027 (3)	−0.003 (2)	0.010 (2)	−0.007 (2)
C11	0.041 (3)	0.018 (3)	0.025 (2)	0.001 (3)	0.012 (2)	−0.002 (2)
C12	0.023 (2)	0.020 (3)	0.028 (3)	−0.001 (2)	0.0047 (19)	−0.002 (2)
C13	0.022 (2)	0.019 (3)	0.029 (2)	−0.005 (2)	−0.0001 (18)	0.001 (2)
C14	0.030 (2)	0.021 (3)	0.029 (2)	−0.004 (2)	0.0031 (19)	−0.003 (2)
C15	0.028 (3)	0.031 (3)	0.040 (3)	−0.004 (3)	−0.004 (2)	0.008 (2)
C16	0.033 (3)	0.025 (3)	0.025 (3)	−0.007 (2)	0.002 (2)	−0.001 (2)
C17	0.038 (3)	0.025 (3)	0.029 (3)	0.003 (3)	0.001 (2)	−0.006 (3)
N1	0.024 (2)	0.022 (2)	0.021 (2)	0.0006 (19)	0.0028 (15)	0.0007 (17)
N2	0.031 (2)	0.015 (2)	0.024 (2)	−0.0043 (19)	0.0019 (17)	−0.0020 (17)
N3	0.031 (2)	0.027 (2)	0.027 (2)	−0.009 (2)	0.0088 (17)	−0.010 (2)
N4	0.041 (3)	0.031 (3)	0.035 (2)	0.003 (3)	0.012 (2)	−0.002 (2)
O1	0.0357 (19)	0.023 (2)	0.0300 (19)	−0.0063 (17)	0.0086 (15)	−0.0027 (16)
O2	0.044 (2)	0.031 (2)	0.0270 (18)	0.002 (2)	0.0030 (15)	−0.0058 (18)
O3	0.0299 (18)	0.0225 (18)	0.0247 (17)	−0.0042 (16)	0.0039 (13)	−0.0062 (15)
O4	0.0305 (18)	0.028 (2)	0.0304 (19)	−0.0073 (18)	0.0109 (15)	−0.0062 (16)

### (II) tert-Butyl N-{(E)-1-[2-(4-cyanobenzylidene)-1-methylhydrazinyl]-3-hydroxy-1-oxopropan-2-yl}carbamate Geometric parameters (Å, º)

**Table:**

C1—C2	1.386 (7)	C11—O2	1.403 (6)
C1—C6	1.422 (7)	C11—H11A	0.9900
C1—H1	0.9500	C11—H11B	0.9900
C2—C3	1.368 (8)	C12—O4	1.208 (5)
C2—H2	0.9500	C12—O3	1.354 (6)
C3—C4	1.405 (8)	C12—N3	1.355 (6)
C3—C17	1.461 (7)	C13—O3	1.481 (6)
C4—C5	1.384 (7)	C13—C14	1.503 (8)
C4—H4	0.9500	C13—C16	1.514 (6)
C5—C6	1.409 (8)	C13—C15	1.529 (7)
C5—H5	0.9500	C14—H14A	0.9800
C6—C7	1.435 (6)	C14—H14B	0.9800
C7—N1	1.286 (6)	C14—H14C	0.9800
C7—H7	0.9500	C15—H15A	0.9800
C8—N2	1.450 (6)	C15—H15B	0.9800
C8—H8A	0.9800	C15—H15C	0.9800
C8—H8B	0.9800	C16—H16A	0.9800
C8—H8C	0.9800	C16—H16B	0.9800
C9—O1	1.219 (6)	C16—H16C	0.9800
C9—N2	1.385 (6)	C17—N4	1.139 (6)
C9—C10	1.524 (7)	N1—N2	1.388 (5)
C10—N3	1.449 (6)	N3—H3	0.8800
C10—C11	1.532 (7)	O2—H2A	0.8400
C10—H10	1.0000		
			
C2—C1—C6	119.9 (5)	C10—C11—H11B	108.7
C2—C1—H1	120.1	H11A—C11—H11B	107.6
C6—C1—H1	120.1	O4—C12—O3	125.9 (5)
C3—C2—C1	120.6 (5)	O4—C12—N3	124.3 (4)
C3—C2—H2	119.7	O3—C12—N3	109.8 (4)
C1—C2—H2	119.7	O3—C13—C14	109.7 (4)
C2—C3—C4	120.7 (4)	O3—C13—C16	103.0 (4)
C2—C3—C17	120.9 (5)	C14—C13—C16	112.3 (4)
C4—C3—C17	118.4 (5)	O3—C13—C15	110.3 (4)
C5—C4—C3	119.8 (5)	C14—C13—C15	111.7 (4)
C5—C4—H4	120.1	C16—C13—C15	109.4 (4)
C3—C4—H4	120.1	C13—C14—H14A	109.5
C4—C5—C6	120.2 (5)	C13—C14—H14B	109.5
C4—C5—H5	119.9	H14A—C14—H14B	109.5
C6—C5—H5	119.9	C13—C14—H14C	109.5
C5—C6—C1	118.8 (4)	H14A—C14—H14C	109.5
C5—C6—C7	122.6 (5)	H14B—C14—H14C	109.5
C1—C6—C7	118.6 (5)	C13—C15—H15A	109.5
N1—C7—C6	120.4 (5)	C13—C15—H15B	109.5
N1—C7—H7	119.8	H15A—C15—H15B	109.5
C6—C7—H7	119.8	C13—C15—H15C	109.5
N2—C8—H8A	109.5	H15A—C15—H15C	109.5
N2—C8—H8B	109.5	H15B—C15—H15C	109.5
H8A—C8—H8B	109.5	C13—C16—H16A	109.5
N2—C8—H8C	109.5	C13—C16—H16B	109.5
H8A—C8—H8C	109.5	H16A—C16—H16B	109.5
H8B—C8—H8C	109.5	C13—C16—H16C	109.5
O1—C9—N2	120.9 (5)	H16A—C16—H16C	109.5
O1—C9—C10	122.3 (4)	H16B—C16—H16C	109.5
N2—C9—C10	116.8 (4)	N4—C17—C3	176.4 (6)
N3—C10—C9	105.5 (4)	C7—N1—N2	118.0 (4)
N3—C10—C11	113.2 (5)	C9—N2—N1	115.8 (4)
C9—C10—C11	109.0 (4)	C9—N2—C8	120.6 (4)
N3—C10—H10	109.7	N1—N2—C8	123.4 (4)
C9—C10—H10	109.7	C12—N3—C10	122.1 (4)
C11—C10—H10	109.7	C12—N3—H3	119.0
O2—C11—C10	114.4 (4)	C10—N3—H3	119.0
O2—C11—H11A	108.7	C11—O2—H2A	109.5
C10—C11—H11A	108.7	C12—O3—C13	121.5 (4)
O2—C11—H11B	108.7		
			
C6—C1—C2—C3	−0.5 (7)	C9—C10—C11—O2	−47.4 (6)
C1—C2—C3—C4	−0.5 (7)	C6—C7—N1—N2	179.5 (4)
C1—C2—C3—C17	178.3 (4)	O1—C9—N2—N1	173.2 (4)
C2—C3—C4—C5	0.0 (7)	C10—C9—N2—N1	−6.9 (6)
C17—C3—C4—C5	−178.9 (4)	O1—C9—N2—C8	−2.4 (7)
C3—C4—C5—C6	1.6 (7)	C10—C9—N2—C8	177.5 (4)
C4—C5—C6—C1	−2.5 (7)	C7—N1—N2—C9	−174.2 (4)
C4—C5—C6—C7	−179.9 (4)	C7—N1—N2—C8	1.2 (7)
C2—C1—C6—C5	2.0 (7)	O4—C12—N3—C10	−14.8 (8)
C2—C1—C6—C7	179.4 (4)	O3—C12—N3—C10	165.8 (5)
C5—C6—C7—N1	5.0 (7)	C9—C10—N3—C12	−150.2 (5)
C1—C6—C7—N1	−172.3 (4)	C11—C10—N3—C12	90.7 (6)
O1—C9—C10—N3	−19.4 (7)	O4—C12—O3—C13	−9.9 (8)
N2—C9—C10—N3	160.7 (4)	N3—C12—O3—C13	169.5 (4)
O1—C9—C10—C11	102.5 (5)	C14—C13—O3—C12	−63.4 (5)
N2—C9—C10—C11	−77.5 (5)	C16—C13—O3—C12	176.8 (4)
N3—C10—C11—O2	69.7 (6)	C15—C13—O3—C12	60.1 (6)

### (II) tert-Butyl N-{(E)-1-[2-(4-cyanobenzylidene)-1-methylhydrazinyl]-3-hydroxy-1-oxopropan-2-yl}carbamate Hydrogen-bond geometry (Å, º)

**Table:**

*D*—H···*A*	*D*—H	H···*A*	*D*···*A*	*D*—H···*A*
N3—H3···O1	0.88	2.27	2.620 (6)	104
O2—H2*A*···O4^i^	0.84	2.09	2.877 (5)	156
C4—H4···N4^ii^	0.95	2.61	3.549 (8)	168

Symmetry codes: (i) *x*+1, *y*, *z*; (ii) −*x*−1, *y*−1/2, −*z*+1.

### (III) tert-Butyl N-[(E)-1-(2-benzylidene-1-methylhydrazinyl)-3-hydroxy-1-oxopropan-2-yl]carbamate Crystal data

**Table:**

C_16_H_23_N_3_O_4_	*F*(000) = 688
*M**_r_* = 321.37	*D*_x_ = 1.257 Mg m^−^^3^
Monoclinic, *P*2_1_/*c*	Mo *K*α radiation, λ = 0.71075 Å
Hall symbol: -P 2ybc	Cell parameters from 3225 reflections
*a* = 10.454 (7) Å	θ = 2.0–27.5°
*b* = 10.571 (7) Å	µ = 0.09 mm^−^^1^
*c* = 15.664 (11) Å	*T* = 100 K
β = 101.172 (12)°	Blade, colourless
*V* = 1698 (2) Å^3^	0.16 × 0.05 × 0.01 mm
*Z* = 4	

### (III) tert-Butyl N-[(E)-1-(2-benzylidene-1-methylhydrazinyl)-3-hydroxy-1-oxopropan-2-yl]carbamate Data collection

**Table:**

Rigaku Mercury CCD diffractometer	2716 reflections with *I* > 2σ(*I*)
Radiation source: fine-focus sealed tube	*R*_int_ = 0.048
Graphite monochromator	θ_max_ = 26.0°, θ_min_ = 2.3°
ω scans	*h* = −11→12
8546 measured reflections	*k* = −13→9
3319 independent reflections	*l* = −19→19

### (III) tert-Butyl N-[(E)-1-(2-benzylidene-1-methylhydrazinyl)-3-hydroxy-1-oxopropan-2-yl]carbamate Refinement

**Table:**

Refinement on *F*^2^	Primary atom site location: structure-invariant direct methods
Least-squares matrix: full	Secondary atom site location: difference Fourier map
*R*[*F*^2^ > 2σ(*F*^2^)] = 0.104	Hydrogen site location: inferred from neighbouring sites
*wR*(*F*^2^) = 0.197	H atoms treated by a mixture of independent and constrained refinement
*S* = 1.23	*w* = 1/[σ^2^(*F*_o_^2^) + (0.0376*P*)^2^ + 3.1065*P*] where *P* = (*F*_o_^2^ + 2*F*_c_^2^)/3
3319 reflections	(Δ/σ)_max_ = 0.002
220 parameters	Δρ_max_ = 0.44 e Å^−^^3^
0 restraints	Δρ_min_ = −0.26 e Å^−^^3^

### (III) tert-Butyl N-[(E)-1-(2-benzylidene-1-methylhydrazinyl)-3-hydroxy-1-oxopropan-2-yl]carbamate Special details

**Table:**

Geometry. All e.s.d.'s (except the e.s.d. in the dihedral angle between two l.s. planes) are estimated using the full covariance matrix. The cell e.s.d.'s are taken into account individually in the estimation of e.s.d.'s in distances, angles and torsion angles; correlations between e.s.d.'s in cell parameters are only used when they are defined by crystal symmetry. An approximate (isotropic) treatment of cell e.s.d.'s is used for estimating e.s.d.'s involving l.s. planes.
Refinement. Refinement of *F*^2^ against ALL reflections. The weighted *R*-factor *wR* and goodness of fit *S* are based on *F*^2^, conventional *R*-factors *R* are based on *F*, with *F* set to zero for negative *F*^2^. The threshold expression of *F*^2^ > σ(*F*^2^) is used only for calculating *R*-factors(gt) *etc*. and is not relevant to the choice of reflections for refinement. *R*-factors based on *F*^2^ are statistically about twice as large as those based on *F*, and *R*- factors based on ALL data will be even larger.

### (III) tert-Butyl N-[(E)-1-(2-benzylidene-1-methylhydrazinyl)-3-hydroxy-1-oxopropan-2-yl]carbamate Fractional atomic coordinates and isotropic or equivalent isotropic displacement parameters (Å^2^)

**Table:**

	*x*	*y*	*z*	*U*_iso_*/*U*_eq_	Occ. (<1)
C1	0.7859 (4)	0.4700 (4)	−0.0477 (3)	0.0348 (9)	
H1	0.7784	0.4009	−0.0872	0.042*	
C2	0.8683 (4)	0.5702 (4)	−0.0568 (3)	0.0412 (11)	
H2	0.9170	0.5695	−0.1021	0.049*	
C3	0.8788 (4)	0.6708 (4)	0.0004 (3)	0.0430 (11)	
H3	0.9349	0.7395	−0.0057	0.052*	
C4	0.8078 (4)	0.6721 (4)	0.0669 (3)	0.0386 (10)	
H4	0.8158	0.7415	0.1061	0.046*	
C5	0.7255 (4)	0.5723 (4)	0.0762 (3)	0.0327 (9)	
H5	0.6769	0.5736	0.1215	0.039*	
C6	0.7141 (4)	0.4698 (4)	0.0188 (2)	0.0311 (9)	
C7	0.6264 (4)	0.3627 (4)	0.0243 (2)	0.0296 (9)	
H7	0.6199	0.2956	−0.0166	0.035*	
C8	0.4643 (4)	0.1543 (4)	0.0243 (3)	0.0344 (9)	
H8A	0.4422	0.1880	−0.0350	0.052*	
H8B	0.3956	0.0962	0.0343	0.052*	
H8C	0.5474	0.1087	0.0317	0.052*	
C9	0.4092 (3)	0.2560 (3)	0.1529 (2)	0.0271 (8)	
C10	0.4332 (4)	0.3650 (4)	0.2184 (2)	0.0285 (8)	
H10	0.4347	0.4465	0.1862	0.034*	
C11	0.5617 (4)	0.3500 (4)	0.2828 (3)	0.0362 (10)	
H11A	0.5651	0.2596	0.2995	0.043*	0.198 (7)
H11B	0.5700	0.4247	0.3256	0.043*	0.802 (7)
H11C	0.6349	0.3651	0.2502	0.043*	
C12	0.2675 (4)	0.4729 (4)	0.2834 (2)	0.0281 (8)	
C13	0.0726 (4)	0.5471 (4)	0.3365 (3)	0.0341 (9)	
C14	−0.0564 (4)	0.4811 (5)	0.3367 (3)	0.0520 (13)	
H14A	−0.0411	0.4045	0.3725	0.078*	
H14B	−0.0970	0.4580	0.2770	0.078*	
H14C	−0.1144	0.5381	0.3607	0.078*	
C15	0.1439 (5)	0.5729 (6)	0.4287 (3)	0.0623 (15)	
H15A	0.1576	0.4932	0.4611	0.093*	
H15B	0.0918	0.6303	0.4574	0.093*	
H15C	0.2285	0.6121	0.4273	0.093*	
C16	0.0522 (5)	0.6640 (5)	0.2808 (4)	0.0669 (16)	
H16A	0.1357	0.7076	0.2838	0.100*	
H16B	−0.0093	0.7204	0.3018	0.100*	
H16C	0.0169	0.6401	0.2204	0.100*	
N1	0.5586 (3)	0.3598 (3)	0.08443 (19)	0.0242 (7)	
N2	0.4760 (3)	0.2584 (3)	0.08675 (19)	0.0266 (7)	
N3	0.3211 (3)	0.3649 (3)	0.2618 (2)	0.0314 (8)	
H1N	0.275 (4)	0.295 (4)	0.251 (3)	0.038*	
O1	0.3346 (3)	0.1683 (2)	0.16179 (18)	0.0340 (7)	
O2A	0.5728 (3)	0.2373 (3)	0.3258 (2)	0.0362 (10)*	0.802 (7)
H2A	0.5770	0.1766	0.2917	0.043*	0.802 (7)
O2B	0.5960 (13)	0.4007 (12)	0.3635 (8)	0.029 (4)	0.198 (7)
H2B	0.5934	0.4800	0.3600	0.035*	0.198 (7)
O3	0.1477 (3)	0.4493 (2)	0.29998 (18)	0.0335 (7)	
O4	0.3204 (3)	0.5758 (2)	0.28835 (19)	0.0383 (7)	

### (III) tert-Butyl N-[(E)-1-(2-benzylidene-1-methylhydrazinyl)-3-hydroxy-1-oxopropan-2-yl]carbamate Atomic displacement parameters (Å^2^)

**Table:**

	*U*^11^	*U*^22^	*U*^33^	*U*^12^	*U*^13^	*U*^23^
C1	0.034 (2)	0.042 (2)	0.029 (2)	0.0071 (18)	0.0076 (17)	0.0056 (18)
C2	0.032 (2)	0.056 (3)	0.038 (2)	0.001 (2)	0.0133 (18)	0.016 (2)
C3	0.039 (2)	0.040 (3)	0.051 (3)	−0.005 (2)	0.012 (2)	0.012 (2)
C4	0.039 (2)	0.035 (2)	0.043 (2)	−0.0030 (19)	0.0095 (19)	0.0015 (19)
C5	0.033 (2)	0.035 (2)	0.032 (2)	0.0011 (17)	0.0091 (17)	0.0036 (18)
C6	0.027 (2)	0.035 (2)	0.031 (2)	0.0035 (17)	0.0064 (16)	0.0084 (18)
C7	0.034 (2)	0.031 (2)	0.0233 (19)	0.0052 (17)	0.0046 (16)	−0.0030 (16)
C8	0.040 (2)	0.027 (2)	0.036 (2)	−0.0023 (17)	0.0084 (18)	−0.0113 (18)
C9	0.031 (2)	0.0163 (18)	0.034 (2)	0.0006 (15)	0.0040 (16)	−0.0021 (15)
C10	0.035 (2)	0.0245 (19)	0.031 (2)	−0.0056 (16)	0.0168 (17)	−0.0027 (16)
C11	0.036 (2)	0.046 (3)	0.028 (2)	−0.0073 (19)	0.0087 (17)	−0.0123 (19)
C12	0.029 (2)	0.028 (2)	0.0270 (19)	0.0001 (16)	0.0057 (15)	−0.0018 (16)
C13	0.034 (2)	0.031 (2)	0.040 (2)	0.0102 (17)	0.0119 (18)	−0.0056 (18)
C14	0.035 (2)	0.052 (3)	0.074 (3)	0.009 (2)	0.021 (2)	−0.011 (3)
C15	0.043 (3)	0.089 (4)	0.057 (3)	0.016 (3)	0.014 (2)	−0.033 (3)
C16	0.063 (3)	0.052 (3)	0.091 (4)	0.026 (3)	0.026 (3)	0.022 (3)
N1	0.0251 (16)	0.0186 (15)	0.0286 (16)	−0.0003 (12)	0.0046 (13)	−0.0010 (13)
N2	0.0318 (17)	0.0237 (16)	0.0242 (16)	−0.0026 (14)	0.0053 (13)	−0.0036 (13)
N3	0.0373 (19)	0.0188 (16)	0.043 (2)	−0.0010 (14)	0.0211 (16)	−0.0022 (15)
O1	0.0369 (16)	0.0228 (14)	0.0443 (17)	−0.0052 (12)	0.0125 (13)	−0.0011 (12)
O2B	0.043 (8)	0.017 (7)	0.027 (7)	0.006 (6)	0.005 (6)	0.001 (5)
O3	0.0355 (15)	0.0233 (14)	0.0465 (16)	0.0034 (12)	0.0200 (13)	−0.0026 (12)
O4	0.0441 (17)	0.0231 (14)	0.0529 (18)	−0.0055 (13)	0.0226 (14)	−0.0072 (13)

### (III) tert-Butyl N-[(E)-1-(2-benzylidene-1-methylhydrazinyl)-3-hydroxy-1-oxopropan-2-yl]carbamate Geometric parameters (Å, º)

**Table:**

C1—C2	1.389 (6)	C11—H11A	0.9900
C1—C6	1.398 (5)	C11—H11B	1.0278
C1—H1	0.9500	C11—H11C	1.0114
C2—C3	1.382 (6)	C12—O4	1.216 (4)
C2—H2	0.9500	C12—N3	1.344 (5)
C3—C4	1.391 (6)	C12—O3	1.350 (4)
C3—H3	0.9500	C13—O3	1.478 (4)
C4—C5	1.386 (5)	C13—C16	1.504 (6)
C4—H4	0.9500	C13—C15	1.516 (6)
C5—C6	1.398 (6)	C13—C14	1.519 (6)
C5—H5	0.9500	C14—H14A	0.9800
C6—C7	1.470 (5)	C14—H14B	0.9800
C7—N1	1.284 (5)	C14—H14C	0.9800
C7—H7	0.9500	C15—H15A	0.9800
C8—N2	1.462 (5)	C15—H15B	0.9800
C8—H8A	0.9800	C15—H15C	0.9800
C8—H8B	0.9800	C16—H16A	0.9800
C8—H8C	0.9800	C16—H16B	0.9800
C9—O1	1.236 (4)	C16—H16C	0.9800
C9—N2	1.358 (5)	N1—N2	1.381 (4)
C9—C10	1.531 (5)	N3—H1N	0.88 (4)
C10—N3	1.465 (5)	O2A—H11A	0.4685
C10—C11	1.523 (5)	O2A—H2A	0.8421
C10—H10	1.0000	O2B—H11B	0.6548
C11—O2B	1.356 (13)	O2B—H2B	0.8400
C11—O2A	1.363 (5)		
			
C2—C1—C6	120.7 (4)	O2B—C11—H11C	108.6
C2—C1—H1	119.6	O2A—C11—H11C	112.7
C6—C1—H1	119.6	C10—C11—H11C	107.8
C3—C2—C1	119.5 (4)	H11A—C11—H11C	107.0
C3—C2—H2	120.2	H11B—C11—H11C	103.4
C1—C2—H2	120.2	O4—C12—N3	124.7 (3)
C2—C3—C4	120.4 (4)	O4—C12—O3	125.4 (3)
C2—C3—H3	119.8	N3—C12—O3	109.8 (3)
C4—C3—H3	119.8	O3—C13—C16	112.1 (3)
C5—C4—C3	120.2 (4)	O3—C13—C15	107.2 (3)
C5—C4—H4	119.9	C16—C13—C15	113.1 (4)
C3—C4—H4	119.9	O3—C13—C14	102.6 (3)
C4—C5—C6	120.0 (4)	C16—C13—C14	110.6 (4)
C4—C5—H5	120.0	C15—C13—C14	110.7 (4)
C6—C5—H5	120.0	C13—C14—H14A	109.5
C1—C6—C5	119.1 (4)	C13—C14—H14B	109.5
C1—C6—C7	118.4 (4)	H14A—C14—H14B	109.5
C5—C6—C7	122.4 (3)	C13—C14—H14C	109.5
N1—C7—C6	120.1 (3)	H14A—C14—H14C	109.5
N1—C7—H7	120.0	H14B—C14—H14C	109.5
C6—C7—H7	120.0	C13—C15—H15A	109.5
N2—C8—H8A	109.5	C13—C15—H15B	109.5
N2—C8—H8B	109.5	H15A—C15—H15B	109.5
H8A—C8—H8B	109.5	C13—C15—H15C	109.5
N2—C8—H8C	109.5	H15A—C15—H15C	109.5
H8A—C8—H8C	109.5	H15B—C15—H15C	109.5
H8B—C8—H8C	109.5	C13—C16—H16A	109.5
O1—C9—N2	121.9 (3)	C13—C16—H16B	109.5
O1—C9—C10	121.0 (3)	H16A—C16—H16B	109.5
N2—C9—C10	117.1 (3)	C13—C16—H16C	109.5
N3—C10—C11	112.0 (3)	H16A—C16—H16C	109.5
N3—C10—C9	105.5 (3)	H16B—C16—H16C	109.5
C11—C10—C9	112.0 (3)	C7—N1—N2	118.3 (3)
N3—C10—H10	109.1	C9—N2—N1	116.8 (3)
C11—C10—H10	109.1	C9—N2—C8	120.5 (3)
C9—C10—H10	109.1	N1—N2—C8	122.5 (3)
O2B—C11—O2A	84.4 (6)	C12—N3—C10	121.8 (3)
O2B—C11—C10	128.1 (6)	C12—N3—H1N	121 (3)
O2A—C11—C10	113.4 (3)	C10—N3—H1N	112 (3)
O2B—C11—H11A	98.4	C11—O2A—H11A	30.8
O2A—C11—H11A	14.0	C11—O2A—H2A	111.3
C10—C11—H11A	104.9	H11A—O2A—H2A	81.1
O2B—C11—H11B	27.8	C11—O2B—H11B	47.0
O2A—C11—H11B	111.2	C11—O2B—H2B	109.4
C10—C11—H11B	107.7	H11B—O2B—H2B	63.3
H11A—C11—H11B	125.2	C12—O3—C13	121.8 (3)
			
C6—C1—C2—C3	−0.2 (6)	C9—C10—C11—O2A	−54.9 (4)
C1—C2—C3—C4	0.1 (6)	C6—C7—N1—N2	179.1 (3)
C2—C3—C4—C5	−0.2 (7)	O1—C9—N2—N1	−178.6 (3)
C3—C4—C5—C6	0.2 (6)	C10—C9—N2—N1	−0.5 (5)
C2—C1—C6—C5	0.3 (6)	O1—C9—N2—C8	−1.7 (5)
C2—C1—C6—C7	178.8 (4)	C10—C9—N2—C8	176.5 (3)
C4—C5—C6—C1	−0.3 (6)	C7—N1—N2—C9	177.7 (3)
C4—C5—C6—C7	−178.7 (4)	C7—N1—N2—C8	0.8 (5)
C1—C6—C7—N1	−179.7 (4)	O4—C12—N3—C10	−17.6 (6)
C5—C6—C7—N1	−1.3 (6)	O3—C12—N3—C10	163.4 (3)
O1—C9—C10—N3	−21.4 (5)	C11—C10—N3—C12	95.9 (4)
N2—C9—C10—N3	160.5 (3)	C9—C10—N3—C12	−141.9 (4)
O1—C9—C10—C11	100.8 (4)	O4—C12—O3—C13	−7.2 (6)
N2—C9—C10—C11	−77.4 (4)	N3—C12—O3—C13	171.8 (3)
N3—C10—C11—O2B	−38.4 (9)	C16—C13—O3—C12	57.0 (5)
C9—C10—C11—O2B	−156.7 (8)	C15—C13—O3—C12	−67.7 (5)
N3—C10—C11—O2A	63.5 (4)	C14—C13—O3—C12	175.7 (3)

### (III) tert-Butyl N-[(E)-1-(2-benzylidene-1-methylhydrazinyl)-3-hydroxy-1-oxopropan-2-yl]carbamate Hydrogen-bond geometry (Å, º)

**Table:**

*D*—H···*A*	*D*—H	H···*A*	*D*···*A*	*D*—H···*A*
N3—H1*N*···O1	0.88 (4)	2.11 (4)	2.623 (4)	116 (3)
O2*A*—H2*A*···O4^i^	0.84	2.09	2.852 (4)	150
O2*B*—H2*B*···O1^ii^	0.84	2.18	2.966 (13)	156
C7—H7···O2*A*^iii^	0.95	2.45	3.229 (5)	140

Symmetry codes: (i) −*x*+1, *y*−1/2, −*z*+1/2; (ii) −*x*+1, *y*+1/2, −*z*+1/2; (iii) *x*, −*y*+1/2, *z*−1/2.
